# Experimental dataset on the effect of soaking time and coagulant type on the overall quality of cheese extracted from Ethiopian belessa-95 (Glycine max) soya bean

**DOI:** 10.1016/j.dib.2020.105841

**Published:** 2020-06-08

**Authors:** Addis Lemessa Jembere

**Affiliations:** Chemical Engineering Program, Faculty of Chemical and Food Engineering, Bahir Dar Institute of Technology**,** Bahir Dar University**,** Bahir Dar**,** Ethiopia

**Keywords:** Soya bean, Soaking time, Coagulant, Soy cheese, Belessa-95

## Abstract

Vegetable protein as a replacement for raw materials for the dairy industries is essential to meet the gap between food supply and demand in Ethiopia with particular accentuation of soy protein-based milk and cheese. The experimental dataset presents the extraction of cheese from the soya bean (Belessa-95 or Glycine max). The effects of soaking time and types of coagulants on the overall properties of extracted soy cheese were analyzed. Sensory evaluation was accompanied to highlight the acceptability of the soy cheese product and to further strengthen the significance difference between samples. The experimental method involved pre-treatment as well as extraction process in which four levels of soaking time was taken (12, 24, 36 and 48 h) and three different types of coagulants were used including lemon juice, vinegar, and CaSO_4_•2H_2_O. Important properties were tasted to evaluate the best possible amount of soaking time and type of coagulant. These properties were mass yield (%), protein content (%), pH and proximate analysis comprising of moisture content (%), ash content (%) and total solid content (%). The different effects eminent within the values of physical tests are a reflection of the diverse coagulants utilized and different soaking time. Sensory analysis was conducted to further investigate the effect of soaking time and coagulant type. Five semi-trained respondents on a five-point hedonic scale were involved in the process. Data gathered from the sensory evaluation were statistically analyzed using one-way analysis of variance (ANOVA) with a significance level of 5%. Principal component analysis (PCA) was performed on the sensory data to provide additional multivariate graphical presentation.

## Specifications Table

**Table:** 

**Subject**	Chemical Engineering
**Specific subject area**	Chemical engineering
**Type of data**	Figures, Tables
**How data were acquired**	Mass yield were determined based on the percentage of the ratio of mass of the cheese to the mass of soya bean. Protein content was calculated based on Kjeldahl method [1], moisture content and Ash content were conducted and recorded as per AOAC (1990) [3]. Total solid content and pH was determined by using AOAC (2000) [4]. Panellists sensory response on different samples were collected and data were analysed with one-way ANOVA using SPSS Statistics version 20 [5], and Principal compnenent analysis (PCA) was carried out using origin Pro 9.0 softwares [6].
**Data format**	Raw and Processed
**Parameters for data collection**	Soy cheese was extracted from a selected Varity soya bean (Belesa-95) by considering 2 factors; these were soaking time at 12, 24, 36 and 48 h and coagulant types including lemon juice, vinegar and CaSO_4_•2H_2_O. Each experiment was conducted on triplicate experimental run. Respondents for sensory evaluation were 5 semi-trained panelists from Bahir Dar Institute of Technology campus.
**Description of data collection**	Soy cheese physical properties related data were collected for all experimental runs in terms of Mass yield, protein content, moisture content, ash content, Total solid content and pH. For all samples response on sensory were evaluated qualitatively on color, odor, taste and firmness. Individulal qualities were rated In five-hendoic scale.
**Data source location**	Pawe agricultural research centre, Pawe District, Ethiopia, Latitude and longitude (and GPS coordinates): 11°19′N and 36°24′E Faculty of Chemical and Food Engineering, Bahir Dar Institute of Technology, Bahir Dar University, Bahir Dar, Ethiopia, Latitude and longitude (and GPS coordinates): 11.5974° N, 37.3960° E
**Data accessibility**	Repository name: **Mendeley Data** Data identification number: 10.17632/sf7pb6dzx2.1 Direct URL to data: https://data.mendeley.com/datasets/sf7pb6dzx2/1

## Value of the Data

• The dataset depicts the effect of some of the most important parameters on the overall quality of soy cheese extracted from selected verity of soya bean, which is useful input for emerging cheese processing industries, there by solving protein-energy malnutrition caused as a result of inflated cost of animal protein particularly encountered in Ethiopia now a days.
• Emerging dietary industries may benefit from this data in terms of giving them the idea of providing alternative nutritive food derived from serials that can potentially resolve dependency of protein obtained from animal.
• Researchers in the area can use this data as preliminary information to further optimize the processes parameters. The data is also important to compare and contrast with other variety of soy bean found elsewhere.

## 1 Data description

The experimental dataset contains data describing quality parameters for cheese extracted from soya bean using three different types of coagulants including lemon juice, Vinegar, and CaSO_4_•2H_2_O at a varied soaking time (12, 24, 36 and 48 h). Data related to sensory-based quality responses are also included.

**Fig. 1:**
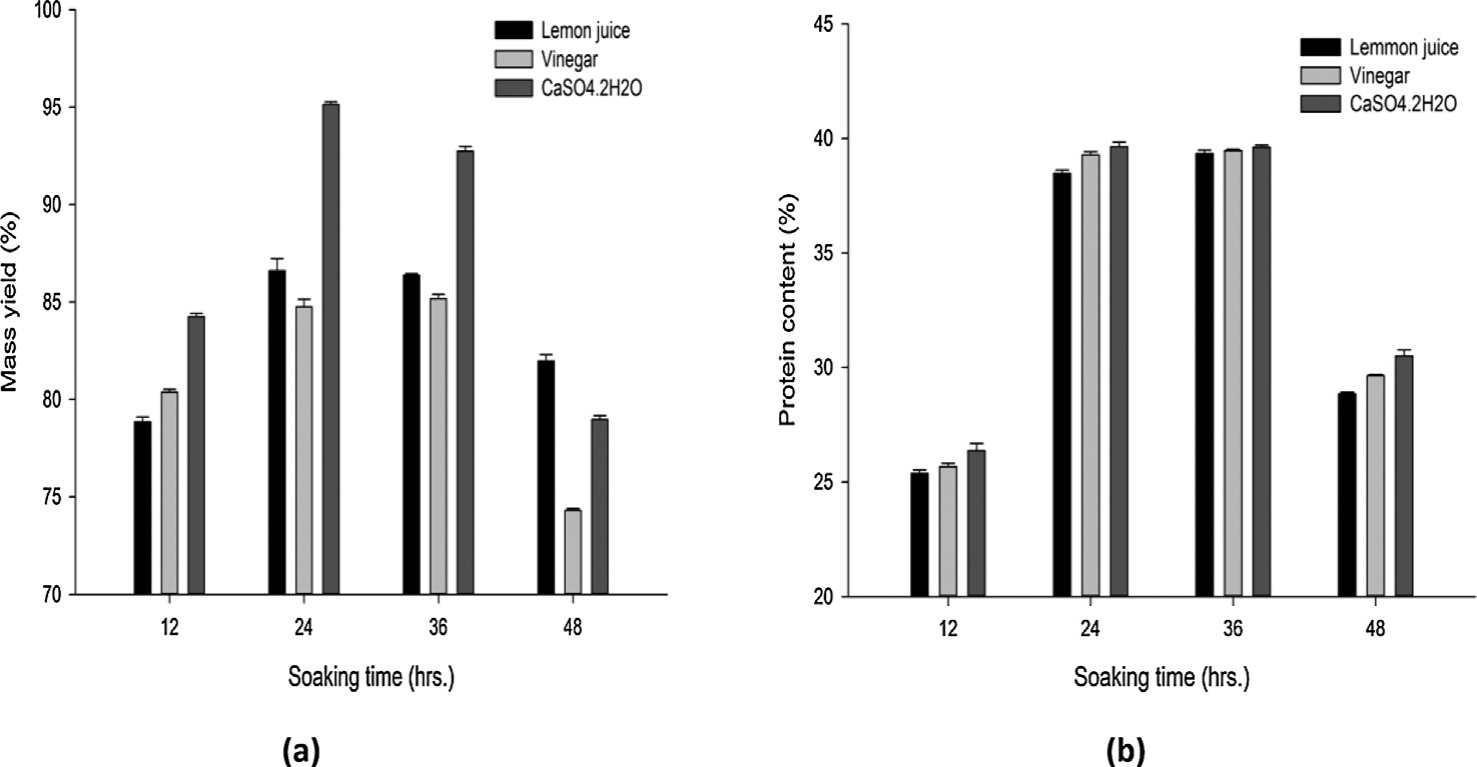
Error bar plot for the Effect of soaking time and types of coagulants on (a) Mass yield and (b) Protein content of soy cheese. Data is expressed as the mean ± standard deviation of replica (*R* = 3) for individual experimental levels (*N* = 12). A Total number of 36 (*R* × *N*) experimental run for every physical property test. Error bars represent standard deviation of the mean. Values calculated using the supplementary material provided as Raw Data-1-DIB-D-20-00914.

**Fig. 2:**
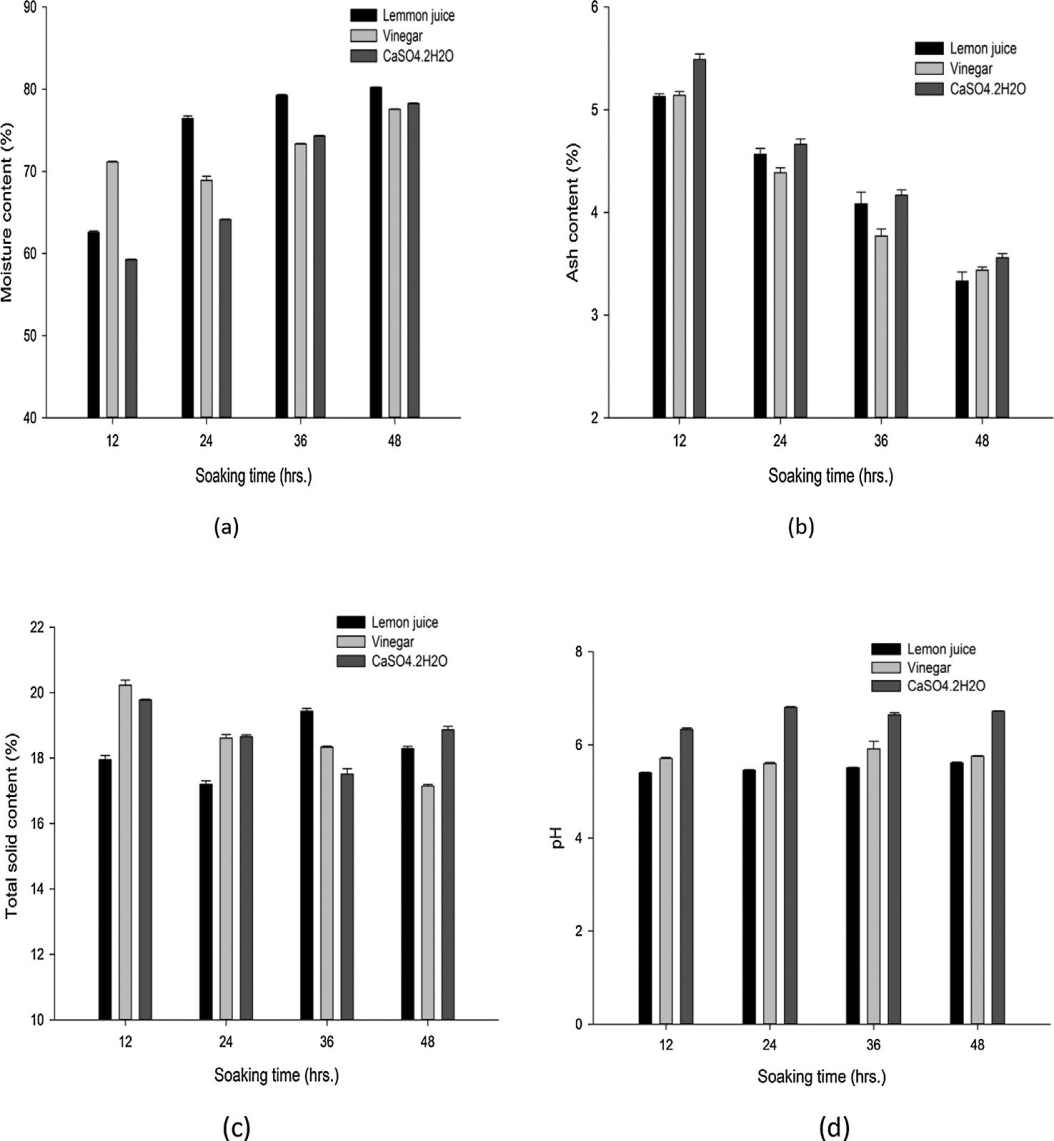
Error bar plot for the Effect of soaking time and types of coagulants on (a) moisture content (b) Ash content (c) Total solid content and (d) pH value of soy cheese. Data is expressed as the mean ± standard deviation of replica experiment (*R* = 3) for individual experimental levels (*n* = 12). A Total number of 36 (*R* × *n*) experimental run for every physical property. Error bars represent standard deviation of the mean. Values calculated using the supplementary material provided as Raw Data-1-DIB-D-20-00914.

**Fig. 3:**
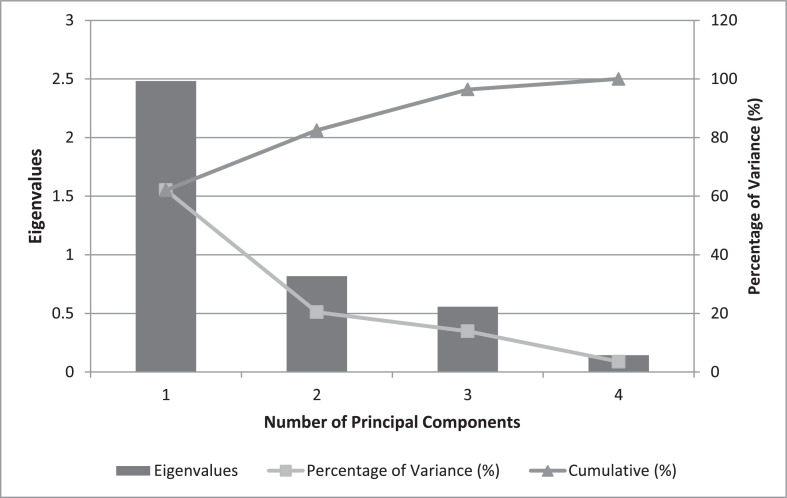
PCA Scree plot of Eigenvalues.

**Fig. 4:**
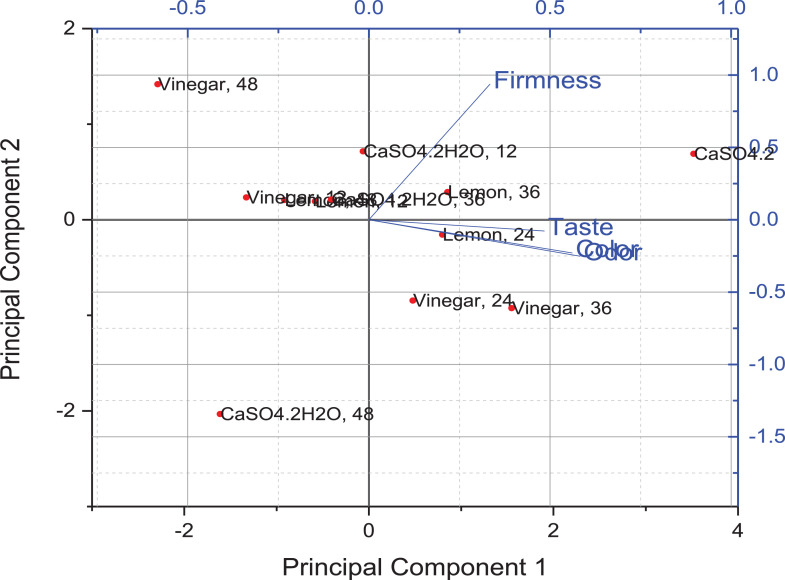
Bi-dimensional plot of panelist variables.

Data comprised in Figs. 1 and 2 shows physical properties that refer to mass yield, protein, moisture, ash and total solid contents and pH values of 12 soya cheese samples, each individual sample content is the mean values ± standard deviations of replica runs (*R* = 3). A total of 36 run were performed for each physical property tests as provided in supplementary materials (File: Raw Data-1-DIB-D-20-00914.xlsx). Fig. 1(a) shows data recorded on the effect of soaking time and coagulant type against soy cheese mass yield. Fig. 1(b) revealed data on the amount of protein content changed with time and diverse coagulant type. Distinctive values of proximate properties were obtained for moisture content (Fig. 2(a)), ash content (Fig. 2(b)), total solid content (Fig. 2(c)) and pH value (Fig. 2(d)). Furthermore, sensory evaluations on different samples were statistically analyzed based on one-way Analysis of Variance (ANOVA) to further highlight the effect of parameters and survey overall acceptability. Qualitative responses on color, odor, taste, and firmness of soy cheese samples were conducted for lemon (Table 1), vinegar (Table 2), and CaSO_4_•2H_2_O (Table 3) coagulants on a five-point hedonic scale. These sensory response values for Individual 12 samples are presented as mean ± standard deviation of responses taken from five semi-trained panelists. The raw data used to analyze the sensory response are given in a supplementary material file named Raw Data-2-DIB-D-20-00914.xlsx. Table 4 contains one way ANOVA that found differences between cheese samples with a significance level of *P* < 0.05, signifying that the panelist were able to sense variances amid diverse samples of soy chees. The mean intensities were compared by Fisher's least square multiple comparison test. Analyzed sensory result is appended as a supplementary material referred as Raw Data-3-DIB-D-20-00914.oxps. PCA was used to establish attribute-sample relationships into some principal components. Fig. 3 shows scree plot of PCA which provide a graphic representation of the quantitative descriptive analysis. PCA generated the four significant principal components (PC1, PC2, PC3 and PC4) that accounted 62.07%, 20.44%, 13.92% and 3.57% of variance respectively with a cumulative contribution rate of more than 60%. Fig. 4 illustrates a Bi-dimensional analysis of the Principal component using the component data matrix shown in Table 5.

**Table 1:** Sensory evaluation result (mean ± standard deviation) on color, odor, taste and firmness for soya cheese samples extracted using lemon coagulant at different soaking time.

Coagulant type	Soaking time(Hrs.)	Color	Odor	Taste	Firmness
Lemon	12	2.6 ± 0.89443	2.8 ± 0.44721	2.6 ± 0.54772	3.2 ± 0.44721
Lemon	24	2.6 ± 0.54772	3.4 ± 0.54772	3.6 ± 0.89443	3.2 ± 0.44721
Lemon	36	3.2 ± 1.09545	3 ± 0.70711	3.2 ± 0.44721	3.6 ± 0.89443
Lemon	48	2.6 ± 0.54772	1.8 ± 0.83666	3.2 ± 1.09545	3 ± 0.70711

**Table 2:** Sensory evaluation result (mean ± standard deviation) on color, odor, taste and firmness for soya cheese samples extracted using Vinegar coagulant at different soaking time.

Coagulant type	Soaking time(Hrs.)	Color	Odor	Taste	Firmness
Vinegar	12	2.4 ± 1.14018	2.2 ± 0.83666	2.6 ± 0.89443	3 ± 0.70711
Vinegar	24	3.6 ± 0.89443	3.4 ± 0.54772	2.4 ± 0.54772	2.8 ± 1.09545
Vinegar	36	3.2 ± 0.44721	3.4 ± 0.54772	4 ± 0.70711	2.8 ± 0.44721
Vinegar	48	1.8 ± 0.44721	1.6 ± 0.54772	2.4 ± 0.54772	3.6 ± 0.89443

**Table 3:** Sensory evaluation result (mean ± standard deviation) on color, odor, taste and firmness for soya cheese samples extracted using CaSO_4_•2H_2_O coagulant at different soaking time.

Coagulant type	Soaking time(Hrs.)	Color	Odor	Taste	Firmness
CaSO_4_•2H_2_O	12	2.6667 ± 0.8165	2.6667 ± 0.8165	3 ± 0.63246	3.6667 ± 0.8165
CaSO_4_•2H_2_O	24	4 ± 0.70711	4.2 ± 0.83666	3.8 ± 0.83666	4.6 ± 0.54772
CaSO_4_•2H_2_O	36	2.4 ± 1.14018	2.8 ± 0.44721	3 ± 0.70711	3.2 ± 0.83666
CaSO_4_•2H_2_O	48	2.5 ± 1	2.5 ± 0.57735	2.75 ± 0.5	1.25 ± 0.5

**Table 4:** Result of ANOVA test with 5% significant level for sensory evaluation of soy cheese samples extracted at different soaking time and types of coagulants.

		Sum of Squares	df	X^2^	F	P
Color	Between Groups	19.667	11	1.788	2.529	.013
	Within Groups	33.933	48	.707		
	Total	53.600	59			
Odor	Between Groups	29.850	11	2.714	6.163	.000
	Within Groups	21.133	48	.440		
	Total	50.983	59			
Taste	Between Groups	15.700	11	1.427	2.724	.008
	Within Groups	25.150	48	.524		
	Total	40.850	59			
Firmness	Between Groups	29.917	11	2.720	5.083	.000
	Within Groups	25.683	48	.535		
	Total	55.600	59			

*Note*: X^2^=Mean square; df =degree of freedom; *F*=Fisher value; *P*=probability value (*P* ≤ 0.05, implies for significance of effect observed).

**Table 5:** Extracted Eigenvector component matrix.

	Component Coefficients			
	PC1	PC2	PC3	PC4
Color	0.56103	−0.22929	−0.45362	0.65338
Odor	0.58272	−0.25155	−0.21942	−0.74096
Taste	0.484	−0.07766	0.8581	0.1529
Firmness	0.33381	0.93708	−0.09877	−0.02636

PC: Principal component.

## 2 Experimental design, materials, and methods

### 2.1 Materials

Raw soya bean required for the experiment was collected from Pawe Agricultural Research Center, Ethiopia. Belesa-95 (Glycine max) variety of soya bean was selected. Hydrated calcium sulfates (CaS0_4_.2H_2_0) was provided from Bahir Dar Institute of Technology. Lemon and vinegar were purchased from local market in Bahir Dar town.

### 2.2 Experimental

As received soya bean sample was subjected to pretreatment steps to remove impurities. 50 g of pretreated soya bean was soaked in 300 ml of water in 500 ml of beaker for a pre-defined soaking time (24–48 h). The hull is removed carefully and the wet soya bean was washed and ground using a lab-scale grinder (WSD-Y-1) at a 1:8 ratio of soya bean to water. Okara which is a byproduct of milk at the end of grinding was separated by the aid vacuum filter. The milk was boiled at 100 °C with an agitation speed of 70 RPM for 45 min. The temperature was subsequently reduced to 70–65 °C. 5% by volume of each coagulant (lemon juice, vinegar, and CaSO_4_•2H_2_O) were added to the milk to obtain an intermediate product curd. The retention time for the coagulant and milk was 15 min. The whey was separated from soya cheese by using a muslin cloth. The produced cheese samples were subjected to different physical tests.

### 2.3 Physical test

Physical properties tested for individual experimental run includes mass yield, protein content, moisture content, Ash content, total solid content and pH. SigmaPlot version 12.5 was used to plot effect of soaking time and coagulant type on the physical properties of soy cheese samples.

#### 2.3.1 Mass yield

The obtained cheese was weighed immediately after maturation. The weights of cheese samples were recorded, and the yield of the cheese was calculated according to the following equation:

(1) $$Mass\;yield\;\left(\%\right)=\frac{mass\;of\;soy\;cheese\;\left(g\right)}{mass\;of\;soya\;bean\;\left(g\right)}\times \;100$$

#### 2.3.2 Protein content

Protein content was determined using an automatic nitrogen determinator (KDN-102F). The approach is based on the Kjeldahl method, a quantitative determination of nitrogen in organic substance plus the nitrogen contained in the inorganic compounds ammonium. Distillation was used to digest the sample at 410 °C in the presence of concentrated H_2_SO_4_ to liberate the reduced nitrogen in the form of ammonium sulfate [1]. Then protein content for soya bean cheese was calculated based on a nitrogen conversion factor of 5.71 [2], expressed as:

(2) Proteincontent(%)=5.71×Nitrogencontent

#### 2.3.3 Proximate tests

##### (a) Moisture content

The moisture content of soya cheese was determined using the oven method AOAC (1990) [3]. 5 g of cheese sample was weighed into pre-weighed aluminum dry dishes and the sample was placed into the dish. The dish and its content were then transferred into the oven at a temperature of 105 °C and dried for 3 h this was then allowed to cool in a desiccator and weighed. The dish was placed back into the oven for another half hour and once more cooled. The process was repeated until a consistent weight was reached. And the moisture content was calculated based on weight loss difference as shown in the equation shown below.

(3) $$Moisture\;content\;\left(\%\right)=\frac{Initial\;weight\;of\;soy\;cheese\;\left(g\right)\;-\;Oven\;dry\;weight\;\left(g\right)\;}{Initial\;weight\;of\;soy\;cheese\;\left(g\right)}\times \;100$$

##### (b) Ash content

AOAC (1990) was adopted to determine ash content present in soya cheese [3]. 5 g of cheese sample was weighed into crucible previously ignited and weighed. The material was ignited in the fume cupboard until no fume was seen charred of organic matter. This was then transferred into muffle furnace at 550 °C using a pair of tongs and was ignited for 3 h’ Followed by cooling in a desiccator, and weighed instantly. Data is recorded using the following relation:

(4) $$Ash\;content\;\left(\%\right)\;=\frac{Initial\;weight\;of\;soy\;cheese\;\left(g\right)\;-\;\text{weight}\;\text{after}\;\text{ignition}\left(g\right)\;}{Initial\;weight\;of\;soy\;cheese\;\left(g\right)}\times \;100$$

##### (c) Total solid content

The total solid content of the different cheese samples was determined according to AOAC (2000) [4]. Three grams of samples were weighed into a dry clean crucible, and then heated in water bath for 15 min. The dish was then placed in an oven at 80 °C overnight (16 h) cooled down in desiccators and weighed. The total solid content was calculated from the following equation:

(5) $$Total\;solid\;content\;\left(\%\right)\;=\frac{weight\;of\;soy\;cheese\;after\;drying}{\;weight\;of\;soy\;cheese\;before\;drying\;\left(g\right)}\times \;100$$

#### 2.3.4 pH

The pH of soya bean cheese samples was determined using digital pH meter (220C). The pH meter was calibrated with buffers of pH 4 and 7. The cheese samples were stirred and the pH value was recorded according to AOAC (2000) [4].

### 2.4 Sensory evaluation

The sensory evaluation of soya cheese carried out by five semi-trained panelists. The samples were evaluated for their color, odor, taste, and firmness. The samples were rated on a five-point hedonic scale to determine overall acceptability (Poor=1, Fair=2, Good=3, Very Good=4, Excellent=5). After the collection of sensory evaluations, Statistical data were generated and analyzed using SPSS Statistics version 20 based on one way ANOVA [5]. Principal Component Analysis (PCA) was analyzed using origin version 9.0.

## Declaration of Competing Interest

The author declare that they have no known competing financial interests or personal relationships which have, or could be perceived to have, influenced the work reported in this article.

## Appendix Supplementary materials

Image, application 1

Image, application 2

Image, application 3

Image, application 4
